# Properties of Rigid Polyurethane Foam Filled with Sawdust from Primary Wood Processing

**DOI:** 10.3390/ma15155361

**Published:** 2022-08-04

**Authors:** Dorota Dukarska, Joanna Walkiewicz, Adam Derkowski, Radosław Mirski

**Affiliations:** Department of Mechanical Wood Technology, Faculty of Forestry and Wood Technology, Poznań University of Life Sciences, Wojska Polskiego 38/42, 60-627 Poznań, Poland

**Keywords:** polyurethane foam, wood by-products, filler, cellular structure, performance properties

## Abstract

In this study, the possibility of using sawdust, a by-product of primary wood processing, as a filler (WF) for rigid polyurethane (PUR) foams was investigated. The effects of the addition of 5, 10, 15 and 20% of WF particles to the polyurethane matrix on the foaming process, cell structure and selected physical-mechanical properties such as density, thermal conductivity, dimensional stability, water absorption, brittleness, compressive and bending strengths were evaluated. Based on the results, it was found that the addition of WF in the amount of up to 10% does not significantly affect the kinetics of the foam foaming process, allowing the reduction of their thermal conductivity, significantly reducing brittleness and maintaining high dimensional stability. On the other hand, such an amount of WF causes a slight decrease in the compressive strength of the foam, a decrease in its bending strength and an increase in water absorption. However, it is important that in spite of the observed decrease in the values of these parameters, the obtained results are satisfactory and consistent with the parameters of insulation materials based on rigid PUR foam, currently available on the market.

## 1. Introduction

In recent years, concern for the environment and sustainable management of renewable resources has become a priority. This trend is clearly visible in the case of the timber industry, for which wood by-products provide an opportunity to expand the resource base but also to produce new materials. Their main source is primary wood processing, but even in highly developed and industrialised countries, the final product in the form of sawn timber placed on the market constitutes only 50% of processed wood. The remaining 50% is material loss, which mainly consists of sawdust and wood chips [1]. The advantage of wood by-products from primary wood processing (sawdust, dust, chips) is that they are free of contaminants that are often found in wood waste harvested from post-consumer wood. These are mainly adhesives, varnishes, paints, metals, glass, plastics, etc. Their presence hinders post-consumer wood recycling processes [2]. Therefore, the use of wood by-products in the manufacturing process of various types of materials is economically and technologically justified. An example of the development of innovative wood-based materials using this type of sorting is chip-sawdust boards or reduced-density cement-chip boards, which can be a substitute for traditional particleboard in the construction industry [3,4,5,6]. It should be noted that there has been for years a growing interest in the use of wood by-products not only in the wood industry but also in the paper industry, the energy industry, and the production of wood–polymer composites.

By-products from both the timber and agricultural industries have also become a source of fillers that can be used in the production of polymer-based composites [7,8]. Wood–Polymer composites (WPCs) combine the advantages of both synthetic polymers and lignocellulosic fillers [9]. Numerous studies conducted in this area have shown that the type, form, shape and particle sizes of the filler significantly affect the properties of WPC composites. In general, it can be stated that the introduction of lignocellulosic filler particles into the polymer matrix reduces the production costs of composites and their weight, provides biodegradability, increases the filling rate, and improves some mechanical properties such as modulus of elasticity or abrasion resistance. Similar results can be obtained in the manufacturing process of composite polyurethane (PUR) foams. Currently, PUR foams account for 2/3 of the world’s polyurethane production and due to their wide range of applications in rigid, semi-rigid and flexible forms, they rank high among all foam materials [10]. In addition to the search for alternative raw materials to polyols and isocyanate, research on the chemical and physical recycling of foams, the modification of the properties of foams by introducing various types of fillers into their structure, has now become one of the main research trends in this area [11].

The advantages of rigid PUR foams include low cost, negligible environmental impact, low density, and high insulation properties (0.018–0.025 W/m·K) [12]. However, they are characterised by low mechanical strength, thermal stability and fire resistance, which may limit the scope of their application [12,13]. The introduction of the optimal amount and type of filler into the foam structure usually improves the physical and mechanical parameters of PUR foams, i.e., their compressive strength, brittleness, thermal resistance, as well as thermal and acoustic insulating properties [12,13,14,15]. The effectiveness of fillers in reinforcing foam structures depends on the number and size of particles, method of incorporation, compatibility with foam components, and degree of dispersion. For these reasons, the use of various types of nanofillers such as: fume silica [16], cellulose nanocrystals [17], spherical TiO_2_, platelet nanoclay, rod-shaped carbon nanofibers [18], montmorillonite [19] and others should be considered as promising solutions in this field. They act as nucleation sites and thus reduce the cell size and, as a result, effectively increase the mechanical properties, fire resistance and thermal stability and reduce the thermal conductivity of PUR foams [13,20]. However, a significant problem with the use of such fillers is their relatively high price and their tendency to agglomerate, which in consequence disturbs the foaming process and deteriorates the properties of the finished foam.

According to Paciorek-Sadowska et al. [21], currently, the modern polyurethane industry is oriented towards environmentally friendly operations. For these reasons, attention has turned to so-called biofillers derived from the processing of natural raw materials. The primary purpose of their use is to improve or maintain the properties of the polyurethane composite while reducing the price of the finished product. The main advantages of biofillers include low acquisition cost, general availability, and an environmentally friendly nature [22]. Moreover, they show the presence of free hydroxyl groups (–OH) capable of reacting with isocyanate groups (–NCO), which gives them sufficient compatibility with polyurethane [23]. The studies carried out so far show that agricultural waste is a material with high application potential in the production of composite PUR foams. It has been shown that waste products such as buckwheat husks [7], walnut shells [12,20], plum stones [22], straw fibre [24], hemp fibre [25], wheat slops [26], rapeseed cake [21], soy and potato protein [27,28], sugar beet pulp [15], egg shell [29], ground coffee [30], keratin chicken feathers [31] and many others have a beneficial effect on the properties of foams. Another source of naturally derived fillers is the timber industry. The suitability of wood waste in the manufacture of PUR composite foams was studied by de Avila Delucis et al. [32], who tested six different forest waste fillers, including wood flour from shavings and sawdust, bark, cones and needles from young pines, kraft lignin and recycled paper sludge from industrial waste. In the course of the study, it was found that the wood floor was the most effective filler among the compositions under study. Rigid PUR foam with its amount of 1 and 5% has the best mechanical and hygroscopic properties, probably due to the higher wood compatibility with the PUR system and the formation of urethane bonds between the filler and isocyanate. Furthermore, according to Yuan and Shi [14], the incorporation of wood flour into the structure of the foam allows it to improve its compressive strength and thermal stability, although it also contributes to a decrease in flexural and tensile strength. Larger admixtures of wood particles to PUR foam (i.e., 10 and 20%), according to Luo et al. [33], may result in a decrease in compressive strength and an increase in water absorption. Augaitisa et al. [23] demonstrated that biocomposite PUR foam with a 0.7 ratio of PUR to pine sawdust has very good physical and insulating properties and also high strength. It is worth emphasising that the examples cited above mainly concern rigid PUR foams with closed-cell structures. However, the authors of this paper have shown in an earlier study [34] that wood by-products sourced from the primary processing of wood can also be a valuable raw material in the production of composite foams with an open-cell structure. It has been demonstrated that a 10 wt% addition of such a biofiller allows them to increase their compressive strength and improve their thermal insulation.

By continuing the research in this field, the investigations were carried out in order to determine the effect of modification of rigid PUR foam with closed-cell structure by different amounts of sawdust obtained by primary wood processing on the kinetics of the foaming process, its structure and selected performance properties. To the best of our knowledge, no research has been conducted to date on the manufacture of closed-cell rigid PUR foams with this type of wood filler, i.e., with this origin and particle size and shape.

## 2. Materials and Methods

### 2.1. Materials

**Figure 1:**
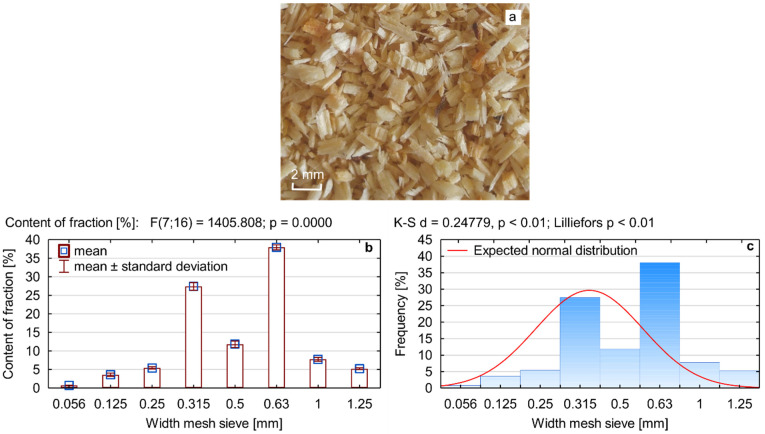
Sawdust used as a filler for PUR foam: (**a**) image, (**b**) fractional composition, (**c**) normal distribution.

A two-component PUREX WG-2732 closed-cell polyurethane thermal insulation system (Polychem System, Poznań, Poland) was used to produce the experimental foams. A major advantage of the system used in this study is that it can be processed manually and by machine, either by injection moulding or pouring. The system includes component A, which is polyol, and component B, which is polymeric 4,4-diphenylmethane diisocyanate (PMDI). Pine sawdust with the largest fraction between 0.315 and 1.25 mm, bulk density of 182 kg/m^3^ and moisture content of about 0.5% were used as foam fillers (WF). This filler, along with its fractional composition and normal distribution, is shown in Figure 1.

These particles were obtained by sorting sawdust generated from primary wood processing. Most of the sawdust from this processing can be successfully used to make chip-sawdust boards; however, the high proportion of fine fractions is always troublesome in the production of this type of panel.

### 2.2. Preparation of PUR/WF Composite Foams for Testing

According to the PUREX WG system manufacturer’s guidelines, the foam components were mixed at a weight ratio of A:B = 100:120. Wood filler was added in amounts of 0, 5, 10, 15 and 20% *w/w* relative to the total weight of the foam components. The entire reaction mixture, including WF, was stirred with a mechanical stirrer at 1200 RPM/min for 10 s at 23 °C and then poured into a metal mould with internal dimensions of 250 × 250 × 130 mm^3^. The foams prepared in this way were cut into samples necessary to determine their physical and mechanical properties. In order to determine the effect of wood filler addition on the foam foaming process, mixtures containing 10 g of component A, 12 g of component B, and an appropriate amount of WF particles were prepared in disposable 400 mL plastic containers.

### 2.3. Characteristics of PUR/WF Composite Foams

The course of foaming of PUR/WF composite foams was characterised on the basis of the duration of successive stages of this process, maximum foaming temperature and measurements of the increase in the height of the foam growth. Thus, creaming times, foam growth start time, gelation and growth time, and tack-free time were measured. The foaming temperature was measured by placing a thermocouple inside the growing foam. Temperature readings were always taken after foam growth was complete, and the readings of the Bench Digital Multimeter (Twintex Electronics Ltd., Shenzhen, China) were stabilised. After the expansion of the foams was completed, the height of the foams was also measured, and the percentage reduction in their growth relative to pure PUR foam was determined on that basis.

The structure of the fabricated foams was visualised using a Hitachi SU3500 (Hitachi, Tokyo, Japan) scanning electron microscope (SEM) at 20× and 60× magnification. A Motic SMZ-168 optical microscope with Motic Images Plus 3.0 software was used to measure cell size and distribution.

The apparent density of foams (ρ), defined as the ratio of the sample mass to its volume, was measured as per EN ISO 845 [35]. Further, 50 × 50 × 50 mm^3^ foams were used for this determination.

The insulating properties of PUR/WF foams were determined using a heat flux density sensor type ALMEMO 117 from Ahlborn (Holzkirchen, Germany) with a wafer dimension of 100 × 30 × 3 mm^3^. Four specimens with dimensions of 230 × 240 × 50 mm^3^ were used for this study. The measured values of heat flux density were used to estimate the average value of thermal conductivity coefficient (λ).

Short-term water absorption (W_p_) tests were conducted on four 200 × 200 mm^2^ samples as per ISO 29767 [36], method A. The foam samples were placed in containers of water in such a position that they were partially submerged in water and their bottom surfaces were 10 ± 2 mm below the water level. After 24 h of soaking, the samples were removed from the water, and dried on a rack at an angle of 45° for 10 min. After this time, the PUR samples were weighed. The value of short-term water absorption of the tested foams was determined as the ratio of the difference in weight of the PUR samples before and after immersion with respect to their surface area.

The dimensional stability of the manufactured foams was determined in accordance with the requirements of EN 1604 [37]. Eight 200 × 200 mm^2^ samples were subjected to conditioning at 23 ± 2 °C and 50 ± 5% relative humidity until equilibrium was reached. The four samples were then placed in a climate chamber at 60 ± 2 °C with a relative humidity of 80 ± 2%. The remaining samples were placed at −20 ± 2 °C. After 24 h of exposure, the percentage changes of their length, width and thickness Δε_l_, Δε_b_, Δε_d_ respectively, were estimated.

Compressive strength tests of the foams (σ_10%_) were conducted 48 h after manufacture and less than 12 h after cutting into specimens. Measurements were performed according to the recommendations of EN 826 [38] using a Tinius Olsen H10KT testing machine (Tinius Olsen Ltd., Salfords, UK). Ten specimens with dimensions of 50 × 50 × 50 mm^3^ were used for this study. The tested foams were compressed at a rate of 5 mm/min in the direction parallel and perpendicular to their growth. The value of the maximum compressive force reached at 10% relative deformation of the foams to their surface was taken as the result.

The three-point bending strength of PUR/WF foams (σ_b_) was determined based on the standard as per EN 12089 [39] on specimens of 130 mm × 40 mm × 20 mm [40], using a Tinius Olsen H10KT testing machine (Tinius Olsen Ltd., Salfords, UK) and a loading rate of 10 mm/min. The average flexural strength of each foam variant tested was determined from ten individual measurements.

The brittleness of PUR/WF foams (K) was determined according to the guidelines of ASTM C 421 [41]. Twelve 25 × 25 × 25 mm^3^ specimens were used and placed in the apparatus chamber along with twelve oak cubes (19 × 19 × 19 mm^3^). The chamber, including samples and oak cubes, was rotated at 60 RPM for 10 min. Brittleness was defined as the percentage weight loss of the test samples. Three trials were performed for each variant.

The yielded test results of composite PUR foams were statistically analysed using STATISTICA software v.13.1 (StatSoft Inc., Tulsa, OK, USA). Mean values of the parameters were compared in a one-factor analysis of variance—post hoc Tukey’s test allowed us to distinguish homogeneous groups of mean values for each parameter for *p* = 0.05.

## 3. Results

### 3.1. Characteristics of the Processing of PUR/WP Foams

The foaming process is an important step that affects the performance of rigid PUR foams [42]. Table 1 presents the results of the processing times of the tested foams and the maximum temperature that was obtained during foaming. It can be concluded that the addition of wood filler causes an increase in the duration time of all foam stages, especially at a higher proportion of WF particles, i.e., 20%. The addition of this amount of filler primarily increases the gelation time by about 36%, the growth time by 40% and the tack-free time by 15%. The extension of these times is a consequence of the deceleration of the exothermic reaction. This is exhibited by the decrease in the maximum foaming temperature by as much as 25 °C, which was observed together with an increase in WF amount. According to the literature on the subject, such unambiguous shaping of the foaming times and temperatures results from the reduced amount of heat released during the reactions taking place in the latent stage (the time from mixing the components to the start of growth) and the foam growth time. This slows down the crosslinking reaction and consequently increases the tack-free time [10,43]. The increase in viscosity of PUR systems under the influence of the filler is also considered to be the main reason for the increase in these times, which consequently limits proper cell expansion. On the basis of studies conducted so far, it can be concluded that with the increase in the number of various types of fillers introduced into the microstructure of foams, higher viscosity of PUR systems and an increase in their processing times are observed [12,15,31,42,44,45,46,47,48,49]. Furthermore, the functional groups of fillers can react with isocyanate groups, which can affect the proper stoichiometry of the reaction and limit the release of blowing agents (CO_2_) [15,44,50]. A consequence of this is the observed reduction in foam expansion as the amount of WF particles introduced into the PUR polymer matrix increases. Due to its bulk density and relatively large particle size, the filler also places a strain on the foam structure, further inhibiting its growth and contributing to the density increase. It was noted that while the 10% and 15% addition of WF causes a reduction in foam height of about 6% and 8%, respectively, the composition containing the maximum amount of WF showed a reduction in the growth of the reaction mixture of about 15%. It is worth noting that such prolongation of foaming times of composite PUR foams produced with various fillers as well as a decrease in maximum temperature of this process is a phenomenon quite commonly reported by researchers. Similar results were obtained in the case of introducing nanoclay, polyester-glass fibre waste, coir fibres, expandable graphite, potato protein, and kraft lignin into the polyurethane matrix [28,43,44,50,51,52].

### 3.2. Structure of PUR/WP Foams

**Figure 2:**
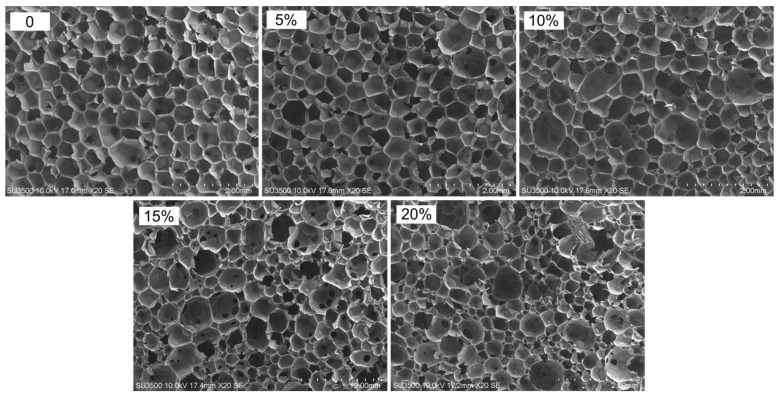
SEM images of PUR foam with different amounts of wood filler.

**Figure 3:**
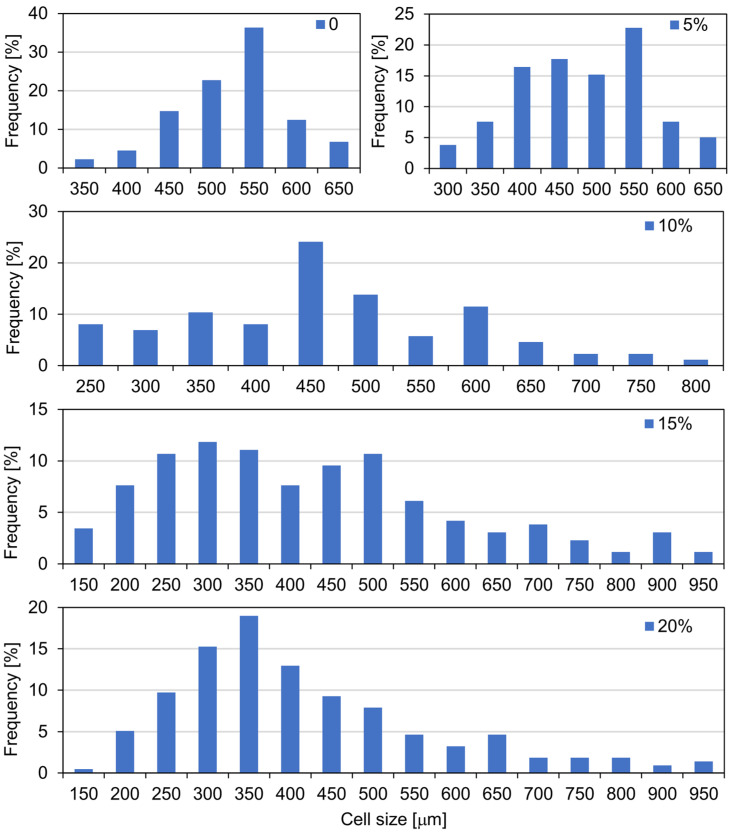
Cell size and cell size distribution of PUR foam depending on wood filler content.

**Figure 4:**
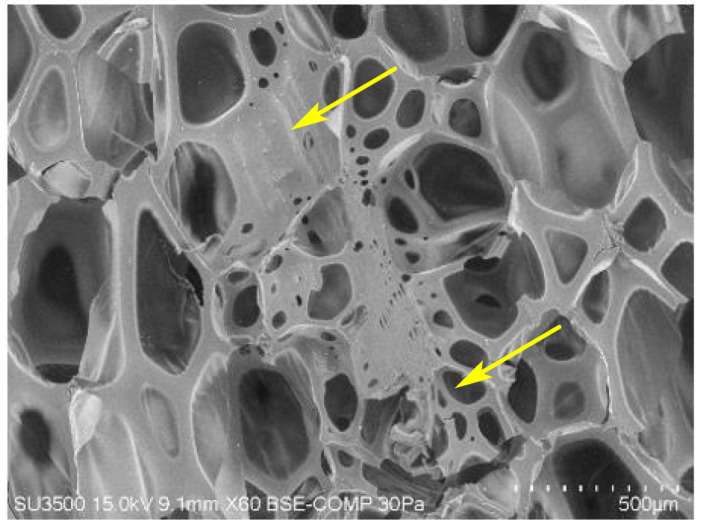
SEM images of PUR foam with addition of wood filler (arrows indicate changes in PUR structure in the presence of wood filler).

The average cell and pore sizes of composite foams are determined, among other things, by microscopic interactions between the polymer matrix and the filler surface [53]. From the SEM micrographs and cell size distribution plots of the experimental foams presented in Figure 2 and Figure 3, it is evident that the pure PUR foam is characterised by a structure with a high content of closed cells with a relatively uniform size distribution, mainly in the range of 450–600 μm. The average size of the foam cells with the highest frequency is 550 μm. However, while the amount of WF particles introduced into the PUR matrix increases, more and more disruption of the foam structure occurs, accompanied by the production of more defective cells. At 5% WF addition, the changes in PUR structure are still relatively small. Increases in smaller cell size were mainly observed in the range of 350–500 μm but also above 550 μm. Further increase in WF admixture results in greater changes in the morphology of the compositions studied. Foams with 10–20% WF have a much less uniform cell shape and a much wider range of cell size distribution. The formation of a larger number of small-sized cells is mainly visible in the range of 200–500 μm, but also cells with sizes far exceeding the cell size of pure PUR foam, namely in the range of 600–950 μm, are evident as well. Significant reduction of the average cell size and disruption of the structure of the produced composite foams can be observed mainly in the interfacial areas, i.e., where the WF particles are clearly attached to the foam cell walls, weakening the cell structure and leading to cracks (Figure 4). An increase in the number of open cells and defective cells with damaged walls and struts is observed. Furthermore, the use of filler particles with larger sizes also causes cell breakage due to incomplete incorporation into the PUR matrix [20,53].

**Figure 5:**
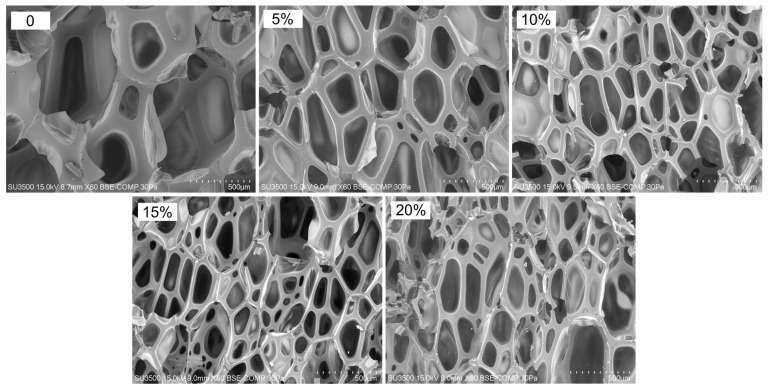
SEM micrographs of PUR showing changes in cell wall structure under the influence of sawdust application.

From the micrographs shown in Figure 5, it can also be concluded that the addition of WF to the PUR matrix causes a reduction in cell wall thickness, which was reflected in the strength test results of the foams. Such observations are confirmed by the work of other authors. The literature shows that the use of virtually any lignocellulosic filler results in weaker cell structures with a large number of open cells, which significantly affects the final properties of polyurethane composites [54]. An unfavourable effect of fillers’ admixture on the morphology of PUR foams is attributed to changes in viscosity and concentration of the reaction mixture, as a result of which the formation and growth of cells are inhibited, leading to a heterogeneous structure of foams [17,55]. In addition, the filler particles attach to the cell walls of the foam, which consequently weakens its structure and leads to destruction [32]. The filler can also cause the nucleation mode to change from homogeneous to heterogeneous and reduce the nucleation energy. The consequence of this is a reduction in cell size in the microstructure of the foams and thus a deterioration in their physical and mechanical parameters [56,57]. Similar changes in the microstructure of PUR composite foams have also been observed with other types of fillers such as walnut shells, plum stones, egg shell waste, sugar beet pulp, sunflower press cake, oak bark, soy protein isolate, talc, carbon nanotubes, graphite and others [12,13,15,22,27,29,31,45].

### 3.3. Apparent Density, Thermal Conductivity and Water Absorption of PUR/WF Foams

**Figure 6:**
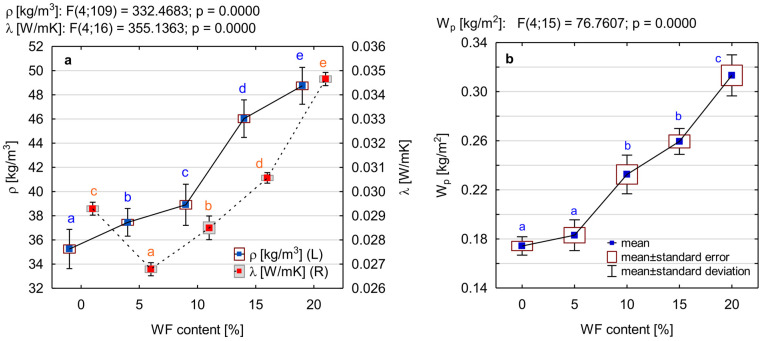
Effect of wood filler addition on: (**a**) apparent density (ρ) and thermal conductivity (λ), (**b**) short-term water absorption by partial immersion (W_p_) of PUR composite foams. Different letters in colour indicate homogeneous groups of mean values determined by one-factor ANOVA with Tukey’s test.

The change in the composition and microstructure of the foams studied was reflected in their physical properties, i.e., density, thermal insulation and surface absorbability determined after short-term immersion. An important characteristic determining the use of foam as an insulating material is its thermal conductivity, the value of which depends, among others, on the apparent density, shape or type of cells (closed or/and open). The data in Figure 6a indicate that the addition of wood filler has a significant effect on the apparent density of the produced foams and on their thermal insulation, which was determined by the thermal conductivity coefficient (λ). This is confirmed by the results of the post hoc test and the different homogeneous groups of mean values of both apparent density and thermal conductivity extracted from it. It is known that the density of composite foams is a function of voids as well as the content and type of filler [31,58]. Since the density of wood filler is greater than that of pure foam, its addition caused a significant increase in the density of PUR/WF foams. This is particularly evident for variants with WF additions above 10%. The apparent density of the pure foam was 35.2 kg/m^3^. The maximum apparent density recorded with 20% filler is 48.7 kg/m^3^, an increase of approximately 38% over pure PUR foam. Such a significant increase in density of foams (especially above 10% WF addition) is an effect of the presence of sawdust in the composition, but also, as suggested by the study of parameters characterising the foaming process, from a significant reduction in their expansion due to an increase in viscosity of PUR systems [15]. The results and conclusions obtained in this respect correlate with many works of other authors who also reported an increase in apparent density of PUR foams due to admixture of various types and amounts of fillers [12,15,20,22,23,52,59].

The consequence of the significant changes in the microstructure and apparent density of the produced composite foams (especially those with higher WF content) are the substitutions of their thermal conductivity. The values of this parameter that were recorded are in the range of 0.0268–0.0347 W/m·K (Figure 6a). Pure PUR foam has a low λ coefficient, i.e., 0.0294 W/m·K. This is close to the values declared by the manufacturers of this type of foam. The introduction of small amounts (5%) of WF into the foam structure resulted in a decrease in thermal conductivity to the level of 0.0267 W/m·K, i.e., by about 10%. According to Kurańska and Prociak [60], the lower value of the thermal conductivity coefficient may be due to an increase in the number of smaller cells and, as a result, cell walls that act as an additional barrier to heat transfer by radiation. A further increase in the WF addition contributes to an increase in thermal conductivity. This is particularly evident in the case of the variant with maximum filler admixture, for which the λ parameter reached values higher than those for pure PUR foam by about 18%. Similar results have been obtained using glass powder, nanoclay, rice straw fibre or walnut shell as PUR foam filler [12,24,51,61]. This is undoubtedly a result of changes in the morphology of the composite foams produced, increased density and increased heat transport by solid parts and thus increased λ_solid_ [19,52]. For closed-cell foams, the increase in thermal conductivity is a consequence of the reduction of closed cells filled with blowing agents, i.e., CO_2_ with a lower thermal conductivity than the conductivity of air present in open cells [19,20]. Despite the recorded increase in the λ coefficient, the obtained results seem to be satisfactory due to the fact all the produced composite foams show a lower thermal conductivity than those commonly used thermal insulation materials, such as polystyrene foam or materials made of mineral wool or wood fibres.

The water resistance of the manufactured foams was determined by examining their susceptibility to short-term soaking after 24 h of partial immersion in water (W_p_). The susceptibility to water absorption of insulation foams, especially foams with fillers of natural origin, is important because it can affect their mechanical properties, thermal conductivity and biological resistance [23]. The main factors affecting the water absorption of foams are the open and closed cell content and their apparent density. In general, rigid PU foams have closed cells and hydrophobic nature. The water, therefore, only penetrated into the pores between the foam cells [62]. As shown in Figure 6b, the addition of wood filler results in a gradual increase in absorption. Statistically significant changes were observed with as little as 10% addition of WF. This is confirmed by the post hoc analysis conducted, which has separated individual homogeneous groups (i.e., b and c) for this amount of WF and higher. A 10% admixture of WF increased the water absorption by approximately 34%, while the maximum amount of filler used in the study (i.e., 20%) increased it by 80%. This significant increase in water absorption for foams with a higher proportion of WF is undoubtedly due to two reasons. Firstly, because of disturbances in foam morphology, i.e., an increase in the number of open cells that are able to store more water [15]. In foams with a structure with fewer closed cells, water migrates more easily than in foams with more closed cells [49]. Additionally, this effect is enhanced by the porous structure of the wood and its hydrophilic nature. This is because wood exhibits the ability to bind water molecules through the active hydroxyl groups of cellulose and hemicelluloses [63]. This is confirmed by studies conducted, among others, by Grząbka-Zasadzińska et al. [54], in which cellulose filler was used to produce PUR foam before and after poly(ethylene glycol) modification. Such an increase in water absorption was also observed when PUR foam was modified with cinnamon or coffee [30,64]. However, it should be emphasised that despite a significant increase in the absorbability of foams, the results obtained (even at 20% addition of WF) are satisfactory and consistent with the results for similar products existing on the market [49].

### 3.4. Dimensional Stability of PUR/WF Foams

**Figure 7:**
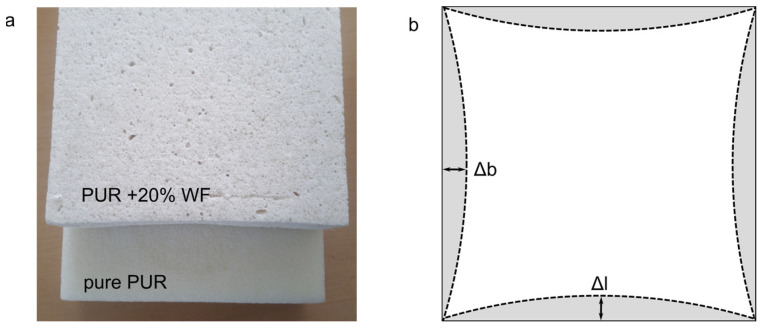
Deformation of pure PUR foam and foam with 20% WF after conditioning: (**a**) images of the tested foams, (**b**) scheme of PUR/WF foam linear dimension changes. Δb, Δl—changes in the width and length of the foams, respectively.

The porous structure, especially the number of closed cells, has a significant effect on two of the most important functional properties of PUR foams, i.e., thermal conductivity and dimensional stability [22]. The dimensional stability of the produced foams was determined after conditioning them for 24 h at elevated temperature and humidity and at a temperature below 0 °C. The obtained measurement results are shown in Table 2. They show that regardless of the exposure conditions, pure PUR foam, as well as foams with WF, added up to 10%, do not show significant changes in dimensional stability. On the contrary, the use of higher amounts of WF, i.e., 15% and 20%, results in a significant deterioration of the dimensional stability of the foams under elevated temperature and relative humidity conditions. In fact, about a 4% reduction in length and width of foams with 15% of wood particles and a 7% change of these dimensions in the case of the variant with 20% WF were observed. Advantageously, irrespective of the amount of filler, no significant changes in the linear dimensions of the foams were observed after the ageing test was carried out at temperatures below 0 °C. It should be noted that in the case of the foam with 20% of WF, significant changes in the linear dimensions (shrinkage) were already observed during its preparation for the ageing tests, i.e., in the time of conditioning at 23 °C and 50% relative humidity required by EN 1604 [37] until it reached the equilibrium state. Even then, the variant showed almost 5% shrinkage in the length and width of the foam, with the largest changes in the middle of their length and width, as can be seen in Figure 7.

However, no significant changes in foam thickness were observed (<0.5%). By Kairytė et al. [65], the increased shrinkage of the foam means that its structure is not rigid enough to provide equilibrium with the pressure difference between the cellular structure and the surrounding environment. The presence of larger closed cells and thinner cell walls, which were observed for foams with a higher proportion of WF, promotes intense diffusion of CO_2_, which increases the shrinkage of the final product [66]. After the ageing tests, this effect is further enhanced by elevated temperature, which accelerates gas diffusion and causes gas expansion, thereby exerting more pressure on the cell walls and consequently changing the volume of the foam [23]. Furthermore, these changes can be attributed to the porous structure and hydrophilic properties of the filler, making it less resistant to wet environments than the hydrophobic polymer matrix. Therefore, the greater its addition, the more negative its effect in this regard on the final product [23].

According to standard EN 13165 [67], for rigid closed-cell foams used as spray insulation, the variation in their length and width after conditioning under elevated temperature and humidity conditions should not exceed 5% and the thickness 10%. By Borkowski [68], in industrial application, polyurethane materials with closed-cell structures should not change their linear dimensions by more than 1–1.5% and geometric volume by more than 3% as a result of ageing. When considering the above requirements, it can be concluded that the foams containing up to 10% wood filler showed a high level of stability. Foams containing higher amounts of WF show significantly lower stability, falling outside the range provided by the above standard.

### 3.5. Compressive and Flexural Properties of PUR/WF Foams

**Figure 8:**
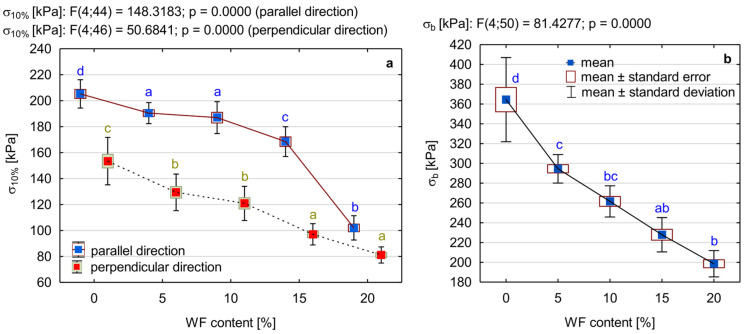
Selected mechanical properties of PUR foam with the addition of different amounts of wood filler: (**a**) compressive strength (σ_10%_), (**b**) flexural strength (σ_b_). Different letters in colour indicate homogeneous groups of mean values determined by one-factor ANOVA with Tukey’s test.

Figure 8 shows the results of selected mechanical properties of the tested PUR/WF foams, i.e., their compressive strength at 10% relative strain (σ_10%_) and flexural strength (σ_b_). Based on them, it was found that despite the increase in apparent density, the introduction of WF particles into the reaction mixture causes a gradual decrease in the compressive strength of the foams, determined both in the direction of growth and perpendicular to it. As expected, higher foam strength values were obtained in the direction parallel to the foam growth, as opposed to the perpendicular direction. These differences are a consequence of the often anisotropic cell structure of PUR foams [69]. For foams with up to 10% WF, the changes in σ_10%_ values are relatively small, at about 10%. A significant decrease in this type of strength was observed at higher amounts of WF, and particularly at 20% addition—the decrease in strength regardless of the direction of foam growth, compared to the reference sample, was 50%. It is well known that increasing the apparent density of foams has a beneficial effect on their mechanical properties, such as flexural strength or compressive strength [70]. The mechanical properties of composite foams are also affected by the type and size of filler particles, surface modification and interaction with the polyurethane matrix [12,69]. There is no doubt that the decrease in compressive strength of PUR/WF foams is a result of disruption of their morphology due to the incremental introduction of wood filler into the polyurethane matrix. As mentioned above, this resulted in more cells with thinner walls, smaller struts, and pronounced cell damage, which is also presented in Figure 4 and Figure 5. Such cells are not strong enough to withstand the compressive load, and more stress is generated on the cell struts [31]. This lack of strengthening effect of PUR foams when filler particles were introduced into their polymer matrix (especially in larger amounts) was also reported in the works of other researchers. For example, a decrease in compressive strength was also obtained when basalt particles, cellulose filler, biochar, polyester composite waste, ground pedunculate oak shoulder, and wheat slops were used [26,42,45,52,69,71]. According to the literature, improvement in compressive strength can be provided by good interfacial adhesion between the filler particles, which are located in the cell struts, and the polyurethane matrix, which facilitates stress transfer. However, a large amount of filler causes cell defects and cell collapse because its particles can pass through the cells causing damage to the foam structure [72]. In the case of the present work, the relatively large particle size of the WF is also important, with approximately 40% of the particles being of the 0.63 mm fraction. Similar to the study conducted by Augaitis et al. [23] (sawdust was used as PUR filler), it was observed that some of the filler particles were not completely incorporated into the polymer matrix. Some of these particles remained loose, indicating insufficient wetting of their surfaces by the PUR matrix, which in turn leads to the formation of voids and open cells in the foam structure [15,23]. The above corresponds to the results of work by Kurańska and Prociak [60], which showed that the filler fibres of shorter lengths are better embedded into the cells of the polymer matrix and do not cause more damage than longer fibres. As a result, foams with longer fibres show lower compressive strength. This is also confirmed by the results obtained by Sture et al. [59], who introduced sawdust in amounts of 0.5–1.5 wt% into the polymer matrix with a significant degree of fineness, i.e., with sizes mainly in the range up to 0.016 mm. No significant structural defects were observed in the final product obtained.

It should be noted that in spite of the recorded decreases in σ_10%_, the compressive strength of the foams containing up to 15% of WF, exceeds the value of 100 kPa (both in the parallel and perpendicular direction to their growth), which is considered sufficient for rigid PUR foams applications [73]. With a WF content of 10%, the requirements for insulating materials (≥120 kPa) are still fulfilled [74]. When considering the above, it can be stated that the results obtained in this respect are satisfactory and consistent with the thermal insulation parameters of materials based on rigid PUR foam, currently available on the market [75].

Similar to the compressive strength, there was a decrease in flexural strength (σ_b_) of the produced PUR/WF foams as the amount of WF increased. While pure PUR foam showed that type of strength at 364 kPa, the introduction of filler in the amount of 20% caused a decrease in the value of σ_b_ to the level at 198 kPa, i.e., by as much as 45%. It should be noted that the first 5% of the filler caused the biggest changes—by about 19%. The reasons for such a significant decrease in flexural strength (as in the case of compressive strength) are changes in the morphology of the foam, the amount of filler and its particle size. In general, foams have better mechanical properties when their cells are intact, uniform in size, evenly distributed, and regular in shape [76]. However, as shown in the SEM images, as the proportion of WF particles increases, the produced foams have smaller cells with thinner walls and struts and increasing damage. The number of open cells also increases. For these reasons, the structure of foams becomes increasingly porous and elastic so that not only the compressive strength but also the flexural strength deteriorates despite the increase in density. Saint-Michel et al. [77] found that the addition of filler can lead to the friability of the PUR cell walls, which consequently promotes rupture instead of deformation. However, the literature on the subject shows that the addition of filler in the right amount and particle size can improve the mechanical properties of PUR foams. In this case, the filler particles present in the polyurethane matrix act as reinforcing centres, generating local stresses under the application of the loading force. This means that during a growing crack, the filler particles cause energy dissipation [12]. When excessive amounts of filler are present (or aggregated), the filler particles cause stress concentrations that promote specimen fracture. This is confirmed by previous studies. For example, the introduction of up to 1% ground walnut shells into PUR foam increases its flexural strength and impact strength. A higher admixture of this filler (5 wt%) led to a decrease in the values of these parameters [12]. Analogous relationships were obtained in the work of Ciecierska et al. [13], in which carbon nanotubes (CNTs) were used as PUR foam fillers. These authors further found that poor adhesion between the foam and filler particles and their uneven distribution in the polymer matrix was also the cause of the deterioration in flexural strength. Strąkowska et al. [15] obtained an improvement in flexural strength by introducing POSS-impregnated sugar beet pulp in the foam in the amount of less than 5 wt%. Similar results were also obtained using dolomite and kaolin at up to 3 wt% and plum stones at 1 and 2 wt% [22,78].

### 3.6. Brittleness of PUR/WF Foams

**Figure 9:**
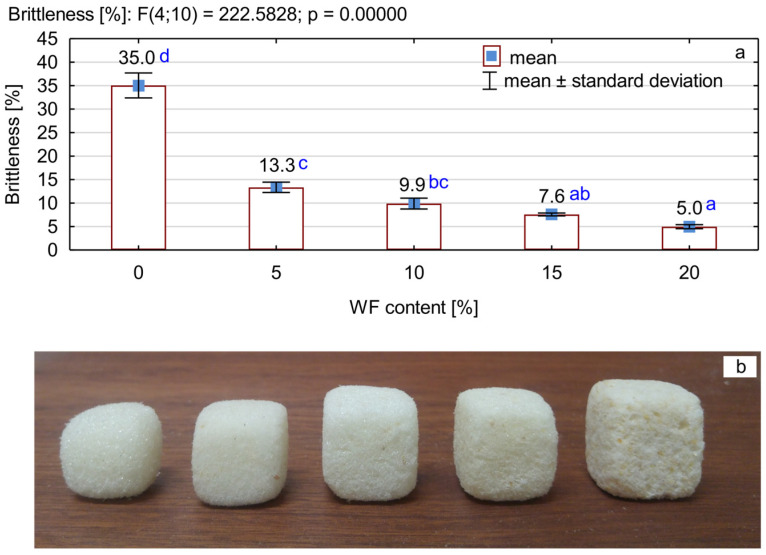
Brittleness of PUR foams with the addition of different amounts of WF: (**a**) average brittleness values of the tested foams, (**b**) image of the foams after the brittleness test (from left to right, an increase in the WF content). Different letters in colour indicate homogeneous groups of mean values determined by one-factor ANOVA with Tukey’s test.

The brittleness of PUR foams is closely related to their compressive strength and microstructure [69]. It was found that the introduction of wood filler particles into the structure of rigid PUR foam significantly reduces its brittleness (Figure 9a,b). The lowest brittleness of about 5% was characteristic for foams produced with the addition of 20% *w/w* of wood particles. This is almost a 6-fold decrease in the brittleness of this type of foam compared to the reference foam. This is usually the result of reducing the size of the enclosed cells, reinforcing the walls with filler and obtaining a more compact structure [21,45]. According to Liszkowska et al. [64,79], the reduction in brittleness due to foam modification is also due to the opening of foam cells and the increase in flexible bonds in the structure of foams.

## 4. Conclusions

Our research shows that it is possible to produce rigid PUR foams with high physical and mechanical properties filled with sawdust, which are a by-product of primary wood (WF) processing. However, in order to produce foams with the required parameters, it is necessary to use an optimal amount of this type of filler.

The research demonstrated that with the increase in WF content, the density of the produced foams increases as well and more and more changes take place in their cellular structure, which significantly affects the formation of their physical and mechanical parameters. The use of this type of filler in the amount of up to 10% does not significantly affect the kinetics of the foaming process. Compared to pure PUR foam, this amount of WF allows to minimise its thermal conductivity and significantly reduces brittleness while maintaining high dimensional stability. However, this results in a slight decrease in the compressive strength of the foam, a decrease in its flexural strength and an increase in water absorption. Despite the observed reduction in the above parameters, the results obtained in this respect are satisfactory and consistent with the thermal insulation parameters of materials based on rigid PUR foam, currently available on the market.

Increasing the content of WF particles in the polyurethane matrix to 15 and 20% results in significant defects in the cellular structure of the foam, which in turn leads to an increase in its thermal conductivity to a level significantly exceeding that of pure PUR foam, a significant decrease in compressive and flexural strength, and an increase in short-term partial water absorption. Foams with such a proportion of WF particles also do not show the required dimensional stability, which practically disqualifies them from application prospects.

It seems that rigid PUR foams containing up to 10% of wood sawdust can be used as thermal insulating materials in the building industry (e.g., door insulation), cooling and heating industry (e.g., insulation of cooling furniture or boilers). Due to its considerably reduced friability, it is also used in the production of usable parts, such as ceiling and wall decorations or polyurethane hives.

When taking into account the results obtained, it is advisable to undertake further work in the direction of improving the properties of PUR/WF foams. This can be achieved by optimising the parameters of the wood filler, which includes the selection of the correct particle size and moisture content. In addition, chemical modification of the surface of the wood filler particles to increase its compatibility with the hydrophobic structure of PUR may be important for improving the properties of foams.

## Figures and Tables

**Table 1:** Parameters characterising the foaming process of PUR/WF foams.

WF Content [%]	Mean Processing Times [s]					T_max_ [°C]	h [mm]	Δh [%]
	Cream	Start of Growth	Gelling	Growth	Tack-Free			
0	12 ± 1 *	38 ± 1	113 ± 6	180 ± 6	249 ± 11	114 ± 2	155 ± 3	-
5	12 ± 1	33 ± 1	122 ± 2	187 ± 3	260 ± 14	113 ± 1	150 ± 2	3.4
10	12 ± 2	30 ± 2	117 ± 4	201 ± 10	258 ± 5	117 ± 2	146 ± 1	5.8
15	11 ± 1	33 ± 2	136 ± 7	240 ± 3	276 ± 6	114 ± 2	143 ± 2	7.7
20	15 ± 2	30 ± 2	157 ± 4	254 ± 6	288 ± 6	90 ± 3	132 ± 9	15.0

*—standard deviation, T_max_—maximum foaming temperature of PUR foam, h—height of the PUR foam growth, Δh—percentage reduction of PUR foam growth.

**Table 2:** Dimensional stability of PUR foam with different amounts of wood filler acclimated under different conditions.

WF Content [%]	T = 60 °C, RH = 80%			T= −20 °C		
	Δε_l_ [%]	Δε_b_ [%]	Δε_d_ [%]	Δε_l_ [%]	Δε_b_ [%]	Δε_d_ [%]
0	0.32 ± 0.09 *	0.37 ± 0.10	0.29 ± 0.11	0.03 ± 0.03	0.05 ± 0.06	0.05 ± 0.08
5	0.32 ± 0.07	0.42 ± 0.14	0.35 ± 0.14	0.03 ± 0.04	0.03 ± 0.04	0.03 ± 0.05
10	1.31 ± 0.28	1.44 ± 0.47	0.24 ± 0.08	0.14 ± 0.07	0.16 ± 0.05	0.01 ± 0.06
15	3.94 ± 0.55	4.05 ± 0.31	0.20 ± 0.10	0.21 ± 0.08	0.23 ± 0.09	0.05 ± 0.05
20	7.53 ± 1.29	6.92 ± 1.40	−0.57 ± 0.26	0.39 ± 0.15	0.42 ± 0.13	0.05 ± 0.08

*—standard deviation, Δε_l_, Δε_b_, Δε_d_—length, width, thickness changes, respectively.

## Data Availability

The data presented in this study are available on request from the corresponding author.
